# Delivery of self-amplifying RNA vaccines in *in vitro* reconstituted virus-like particles

**DOI:** 10.1371/journal.pone.0215031

**Published:** 2019-06-04

**Authors:** Adam Biddlecome, Habtom H. Habte, Katherine M. McGrath, Sharmila Sambanthamoorthy, Melanie Wurm, Martina M. Sykora, Charles M. Knobler, Ivo C. Lorenz, Marcio Lasaro, Knut Elbers, William M. Gelbart

**Affiliations:** 1 Department of Chemistry and Biochemistry, University of California, Los Angeles, California, United States of America; 2 Boehringer Ingelheim Pharmaceuticals, Ridgefield, Connecticut, United States of America; 3 Boehringer-Ingelheim RCV GmbH & Co KG, Vienna, Austria; 4 Tri-Institutional Therapeutics Discovery Institute, New York, New York, United States of America; 5 Boehringer-Ingelheim Pharma GmbH & Co KG, Biberach, Germany; University of California San Francisco, UNITED STATES

## Abstract

Many mRNA-based vaccines have been investigated for their specific potential to activate dendritic cells (DCs), the highly-specialized antigen-presenting cells of the immune system that play a key role in inducing effective CD4^+^ and CD8^+^ T-cell responses. In this paper we report a new vaccine/gene delivery platform that demonstrates the benefits of using a self-amplifying (“replicon”) mRNA that is protected in a viral-protein capsid. Purified capsid protein from the plant virus *Cowpea Chlorotic Mottle Virus* (CCMV) is used to *in vitro* assemble monodisperse virus-like particles (VLPs) containing reporter proteins (e.g., Luciferase or eYFP) or the tandem-repeat model antigen SIINFEKL in RNA gene form, coupled to the RNA-dependent RNA polymerase from the *Nodamura* insect virus. Incubation of immature DCs with these VLPs results in increased activation of maturation markers – CD80, CD86 and MHC-II – and enhanced RNA replication levels, relative to incubation with unpackaged replicon mRNA. Higher RNA uptake/replication and enhanced DC activation were detected in a dose-dependent manner when the CCMV-VLPs were pre-incubated with anti-CCMV antibodies. In all experiments the expression of maturation markers correlates with the RNA levels of the DCs. Overall, these studies demonstrate that: VLP protection enhances mRNA uptake by DCs; coupling replicons to the gene of interest increases RNA and protein levels in the cell; and the presence of anti-VLP antibodies enhances mRNA levels and activation of DCs *in vitro*. Finally, preliminary in vivo experiments involving mouse vaccinations with SIINFEKL-replicon VLPs indicate a small but significant increase in antigen-specific T cells that are doubly positive for IFN and TFN induction.

## Introduction

The delivery of RNA genes has great potential in a range of therapeutic applications[1–5], with the advantage – compared to DNA gene delivery – that there is no nuclear localization and thus no possibility of genomic integration. Some disadvantages it poses for gene delivery include its vulnerability to ribonuclease (RNase) digestion and – in the case of non-vaccine genes – its triggering of innate immune responses[6,7]. Another limitation for *in vivo* applications is that gene expression in targeted cells does not have any amplification, resulting in transient and low expression levels. Accordingly, a gene delivery platform that includes *self-amplifying* mRNA inside a *protective* capsid allowing for cell targeting and uptake[8–11] could represent a major step forward in mRNA-based gene therapy. We address these issues by using viral replicons (self-replicating RNA molecules) for the self-amplification, and *in vitro* self-assembled virus-like particles (VLPs) for the protection, specifically using the RNA-dependent RNA polymerase (RdRp) from *Nodamura virus* (NoV) and capsid protein from *Cowpea Chlorotic Mottle virus* (CCMV).

NoV is a positive-sense RNA insect virus with a bipartite genome whose two molecules are co-packaged in the same virion[12]. The larger RNA molecule includes the RNA1 [≈3200 nucleotides (nt)] gene that encodes for the RdRp, and a subgenomic RNA3 (≈400 nt) encoding the B2 protein that suppresses host-cell RNA interference[13]. The other molecule is the (≈1350 nt) RNA2 that encodes the capsid protein. In addition to replicating in natural insect hosts such as Drosophila, NoV has been shown to also have strong RdRp-dependent replication in mammalian cells[14]. Further, it has been demonstrated that – not only its own genes - but also any gene of interest can be amplified if inserted into the subgenomic region of RNA1 directly after the RdRp open reading frame and before the 3’ untranslated region (UTR)[15].

CCMV is a positive-sense RNA plant virus with a tripartite genome of four genes contained in three single-stranded RNA (ssRNA) molecules[16]. Like NoV, CCMV is a spherical, icosahedral virus whose capsid has a Caspar-Klug triangulation number of 3[17]: each of the CCMV ssRNAs is separately packaged in a T=3 shell of 180 subunits, organized as 12 pentamers and 20 hexamers of a single capsid protein[16]. It has been demonstrated that the CCMV capsid protein can package any of a large variety of heterologous ssRNA *in vitro* into wildtype capsids, as long as the length lies in the range 2500-4200nt so that it does not significantly differ from that (3200nt) of the largest of the CCMV RNAs[18–21]. These *in vitro* assembled capsids, known as virus-like particles (VLPs) – and in particular ones containing RNA replicons – have been shown[22] to both lend protection to the encapsulated RNA when incubated with RNases, and make available its genetic cargo to translation upon delivery to mammalian cells. Because of the unique ability of CCMV capsid protein to package heterologous RNA into perfectly-monodisperse icosahedrally-symmetric (26-nm/180-protein) nanoparticles[18–21], the virus-like particles we use as self-replicating gene-delivery vectors are uniquely well-characterized. Similar results have been demonstrated with cylindrical VLPs reconstituted with capsid protein from *Tobacco Mosaic Virus* (TMV) and RNA replicons from *Semliki Forest Virus*, with the replicons requiring that a TMV “origin-of-assembly”/packaging sequence be inserted into them[23].

**Fig 1:**
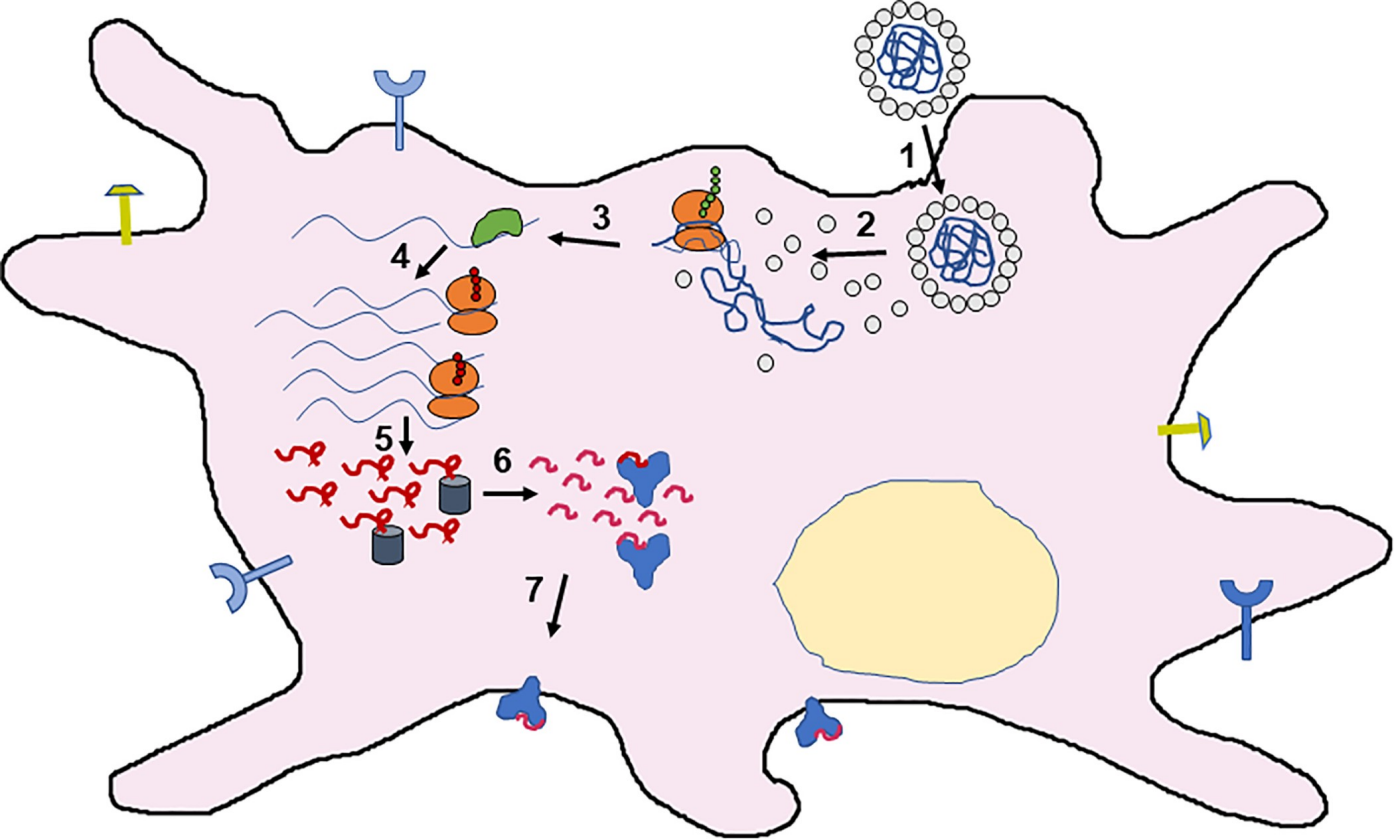
Schematic of DC activation triggered by VLP delivery of a self-replicating vaccine. Upon uptake (1), disassembly (2) of the VLP makes its RNA gene content accessible to the ribosomal machinery (red small and large subunits). Of the two protein gene products – the RdRp of the Nodamura virus, and the tandem OVA SIINFEKL epitope repeats – only the RdRp (green blob) is shown (3). There follow (4) many rounds of replicon amplification (by the RdRp) and translation of OVA repeats (long red squiggles), and subsequent processing (5) of the repeats by proteasomes (grey cylinders) and charging (6) of MHC-I molecules (blue Ys) with individual SIINFEKL epitopes (short red squiggles), which are then secreted (7) for presentation at the plasma membrane, along with CD 80, 86, and MHC-II molecules (lollipops).

It is a feature of our replicon gene delivery platform that the gene of interest is coupled to the replication and translation of the NoV viral components, so that it has the capability of being used for a large array of possible therapies. Here we use the reporter proteins luciferase and enhanced yellow fluorescent protein (eYFP) to characterize and quantify the coupling of RNA replication and protein expression by the replicon. We then evaluate the efficacy of this platform for delivering antigens in RNA form by incorporating into the replicon a model antigen sequence (Ovalbumin epitope SIINFEKL[24]). Since many immune cells are naturally primed to detect foreign virus particles and take them up by endocytosis, the targeting problem is already partially solved for these VLPs. The sustained amplification of the antigen sequence by the replicon leads to increased translation of the model antigen, and this allows for greater marker activation and Ovalbumin antigen presentation by the major histocompatibility complex I (MHC I) of the immune cell – see Fig 1.

In this paper we demonstrate that amplification of reporter genes by the NoV replicase results in much greater protein expression than an equal copy number of the reporter gene mRNA alone. Further, when immature DCs are incubated with VLPs containing this replicon mRNA significant increases in DC maturation and RNA levels of the gene of interest (eYFP or the tandem OVA SIINFEKL epitope) are observed. The samples with *un*packaged mRNA or replicon mRNA show no perceptible change in maturation or RNA levels compared to negative controls, pointing up the fact that the VLP effectively protects the mRNA and helps it gain entry into the DCs.

Because many vaccines need to be administered incrementally in doses, an additional important factor is how the drug/vaccine is affected by existing immunity against the exposed outer portion of VLPs. Accordingly, antibodies were generated against CCMV capsid protein (CP) by immunizing mice with CCMV VLPs containing a non-translatable RNA. VLPs containing eYFP-replicon or OVA-replicon were incubated with these polyclonal anti-CCMV CP antibodies before being added to immature DCs, leading to enhanced DC maturation and RNA levels of eYFP or OVA compared to those of “naïve” VLPs, i.e., ones that have not been exposed to antibodies.

Finally, in preliminary in vivo experiments, we have measured the T-cell response to a series of mouse vaccinations with VLPs containing the SIINFEKL antigen in replicon form, and find a small but significant population of SIINFEKL-sensitive T cells that are doubly-positive in INF and TFN cytokine production.

## Results and discussion

### Hijacking of the Nodamura virus life cycle

**Fig 2:**
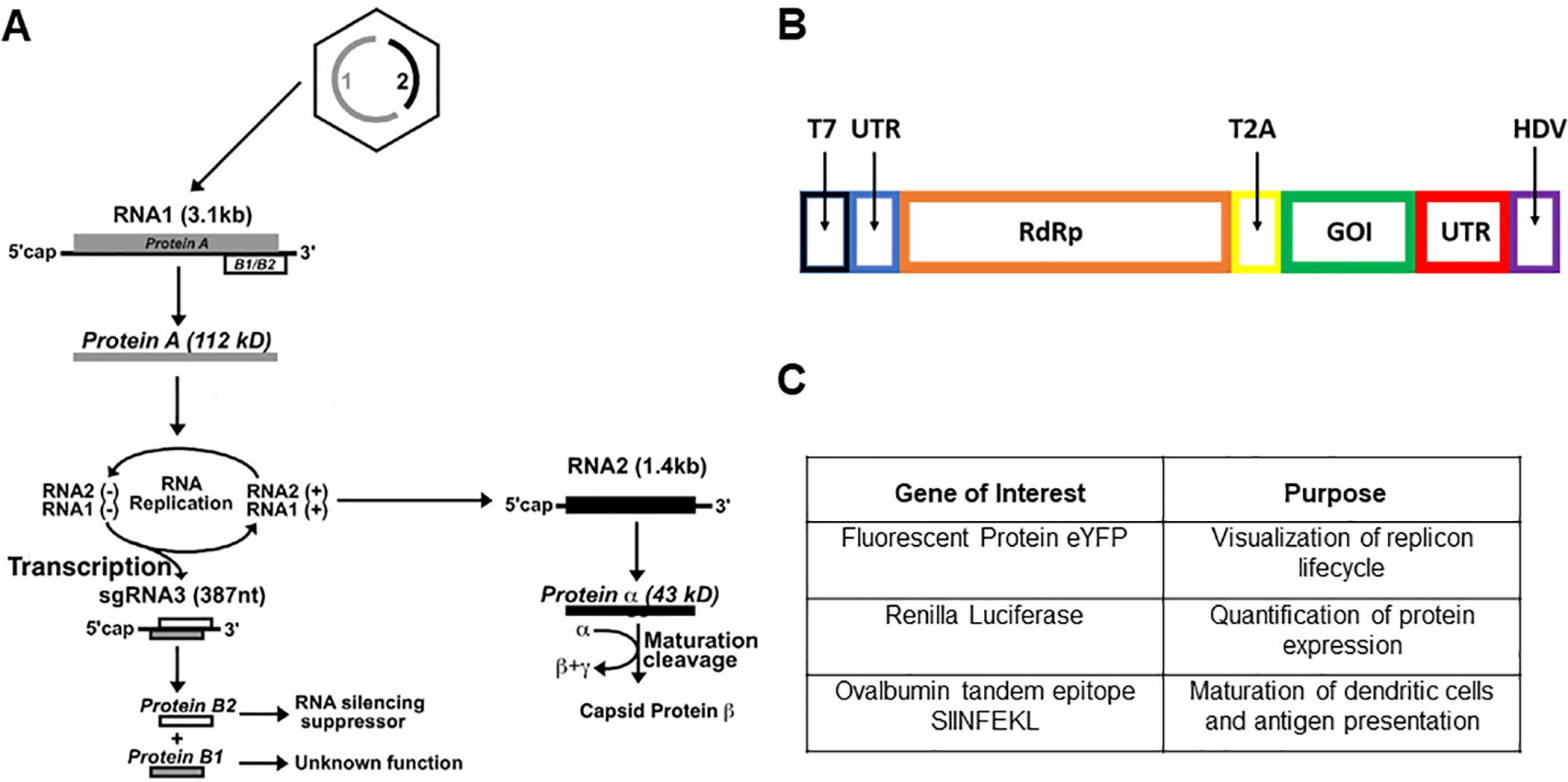
A Protein synthesis in the lifecycle (see discussion in the text) of NoV[25]. **B Schematic of the replicon construct used to amplify and express an arbitrary gene of interest (GOI)**. T7 is the transcriptional promoter, and the left and right UTRs are the 5’ and 3’ untranslated regions, respectively, of NoV RNA1 that are needed for replication. “RdRp” is the NoV RNA-dependent RNA polymerase. T2A is *Thosea asigna* virus 2A self-cleaving peptide that allows the RdRp-GOI polyprotein to function as two independent proteins, subsequent to translation. HDV is the Hepatitis Delta Virus ribozyme for ensuring clean RNA transcripts. **C** Table of genes of interest, inserted one at a time into the replicon depicted in B.

Fig 2A, which is adapted from Ball, et al.[24] shows the protein synthesis components of the NoV life cycle, depicting schematically the replication and translation strategies of this two-molecule/four-gene positive-sense RNA virus. Molecule RNA1 is translated directly to yield “Protein A”, the RdRp that binds RNA1 and RNA2 and replicates them strongly through successive rounds of -strand and +strand synthesis. As part of the replication of RNA1 its -strands are “transcribed” to give not only +strands of the full-length RNA1, but also mRNA (subgenomic [sg] RNA3) that is translated to either a B1 protein in the same reading frame as the RdRp, or a B2 protein in an alternate reading frame. The B2 protein is known to suppress host-cell RNA interference[14], whereas the function of B1 has not yet been determined. RNA2 is translated to capsid protein alpha, which assembles into a capsid in which the protein matures by undergoing autocatalytic cleavage to yield capsid protein beta and the membrane-permeabilizing peptide gamma.

In the present work we dispense with RNA2 and add a gene of interest (GOI) to the subgenomic sequence of RNA (see METHODS), as shown in Fig 2B. Any gene of interest can be coupled to the replication of the RdRp by inserting it in this way, but its length must be less than about 1000 nt in order to guarantee *in vitro* packaging[18–21] of the ensuing replicon by CCMV CP into a single RNase-resistant wildtype (T=3) capsid. This GOI-containing replicon is constructed in a plasmid that can be transcribed *in vitro*, and includes other important features such as a self-cleaving peptide sequence (T2A peptide) derived from *Thosea asigna virus*, which separates the viral and exogenous proteins to render them functional, and a Hepatitis-Delta-Virus (HDV) ribozyme for providing monodisperse RNA transcripts.

Depending on the goal of our assay, we use one of several GOIs (see table in Fig 2C): eYFP for direct qualitative visualization of replicon replication and translation; Renilla Luciferase for quantification of the resulting level of protein expression from the replicon; and tandem repeats of the SIINFEKL epitope of Ovalbumin for quantification of DC maturation (marker protein expression) and antigen presentation, and for future follow-up *in vivo* experiments assaying T-cell response in mice.

### Reporter genes in replicon form are strongly replicated and expressed

**Fig 3:**
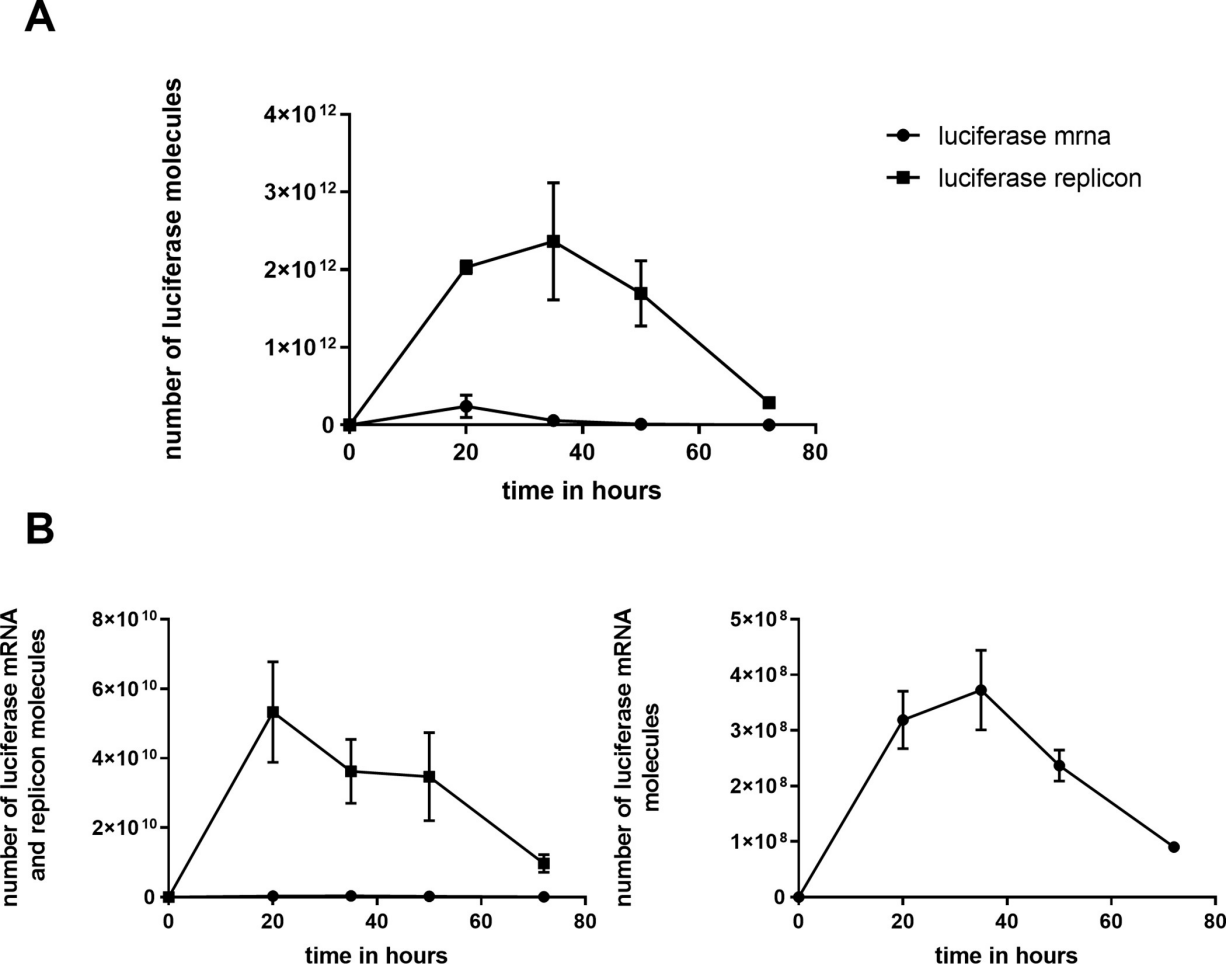
A Time course of luciferase expression in BHK-21 cells. Cells were transfected with equal numbers of renilla luciferase gene, in mRNA form (“luciferase [luc] mRNA”) – including a poly-A tail – or as NoV replicon with luciferase in the sub-genomic region (“luciferase replicon [luc-rep] RNA”). The plot shows the estimated number of luciferase molecules as determined using the standard curves from the Promega renilla luciferase activity kit. **B Number of luc mRNA (solid circles) and luc-rep RNA (solid squares) molecules at each of the time points in A**. These numbers were determined by real-time quantitative PCR (qPCR), with results for both luciferase activity and qPCR from biological duplicates. Each of those biological duplicates was measured in duplicate or triplicate for luciferase activity and qPCR, respectively. Of these replicates, the average is plotted with standard deviation (in some cases the error bar is narrower than the data point itself, thus is not included). The figure on the right is a re-plotting – with an expanded scale in the units of 10^8^ instead of 10^10^ – of the mRNA numbers that are indistinguishable from zero in the figure on the left.

Fig 3A shows the time courses for luciferase protein yield from baby hamster kidney (BHK) cells transfected by lipofectamine with replicon versus naked mRNA forms of luciferase, emphasizing the 30-fold greater yield found for the replicon, and its persistence over several days. Along with the 30-fold greater yield of luciferase molecules, a 150-fold increase in RNA molecules including the luciferase gene is observed with the replicon (Fig 3B).

### Nodamura replicons can be *in vitro* packaged into and protected by virus-like particles

**Fig 4:**
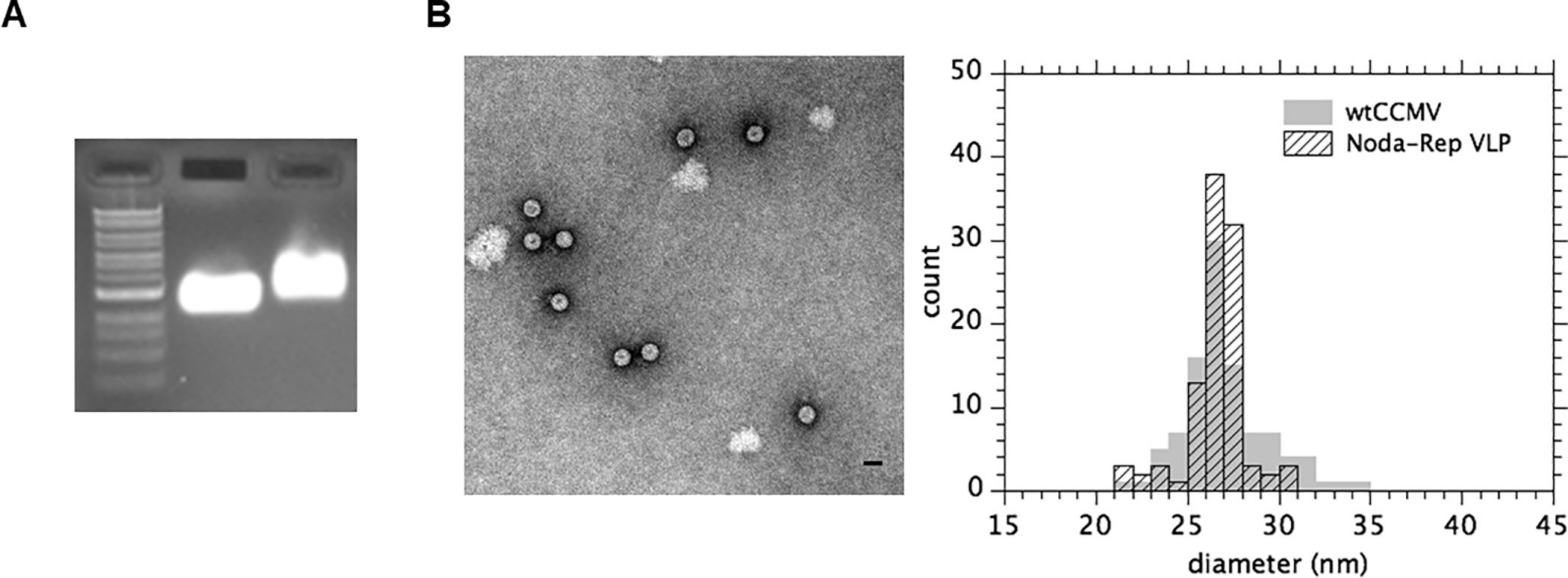
Verification of replicon VLP assemblies. **A)** A native 1% agarose gel with (left to right) a NEB 1 kb DNA ladder, wild-type CCMV virion, and reconstituted VLPs containing NoV replicon RNA.**B) LEFT.** Negative-stain electron microscopy (EM) image of *in vitro* self-assembled VLPs containing eYFP replicon mRNA. **B) RIGHT**. Histogram of size distribution of VLPs shown in the electron micrograph, juxtaposed on the corresponding histogram of sizes for wildtype CCMV virions.

Fig 4A and 4B show gel and electron micrograph images and size histograms, respectively, of *in vitro* reconstituted VLPs prepared (see METHODS) from purified CCMV CP and *in vitro* transcribed eYFP replicon RNA. All figures are for samples that have been treated with RNase, confirming that the VLPs are able to protect RNA cargo. Note that the reconstituted VLPs run a little slower than the wildtype virions because they are prepared from a small excess of protein (see discussion in Materials and Methods) that binds to the outside of the self-assembled capsid.

### DCs maturation and RNA replication are strongest for packaged replicons

**Fig 5:**
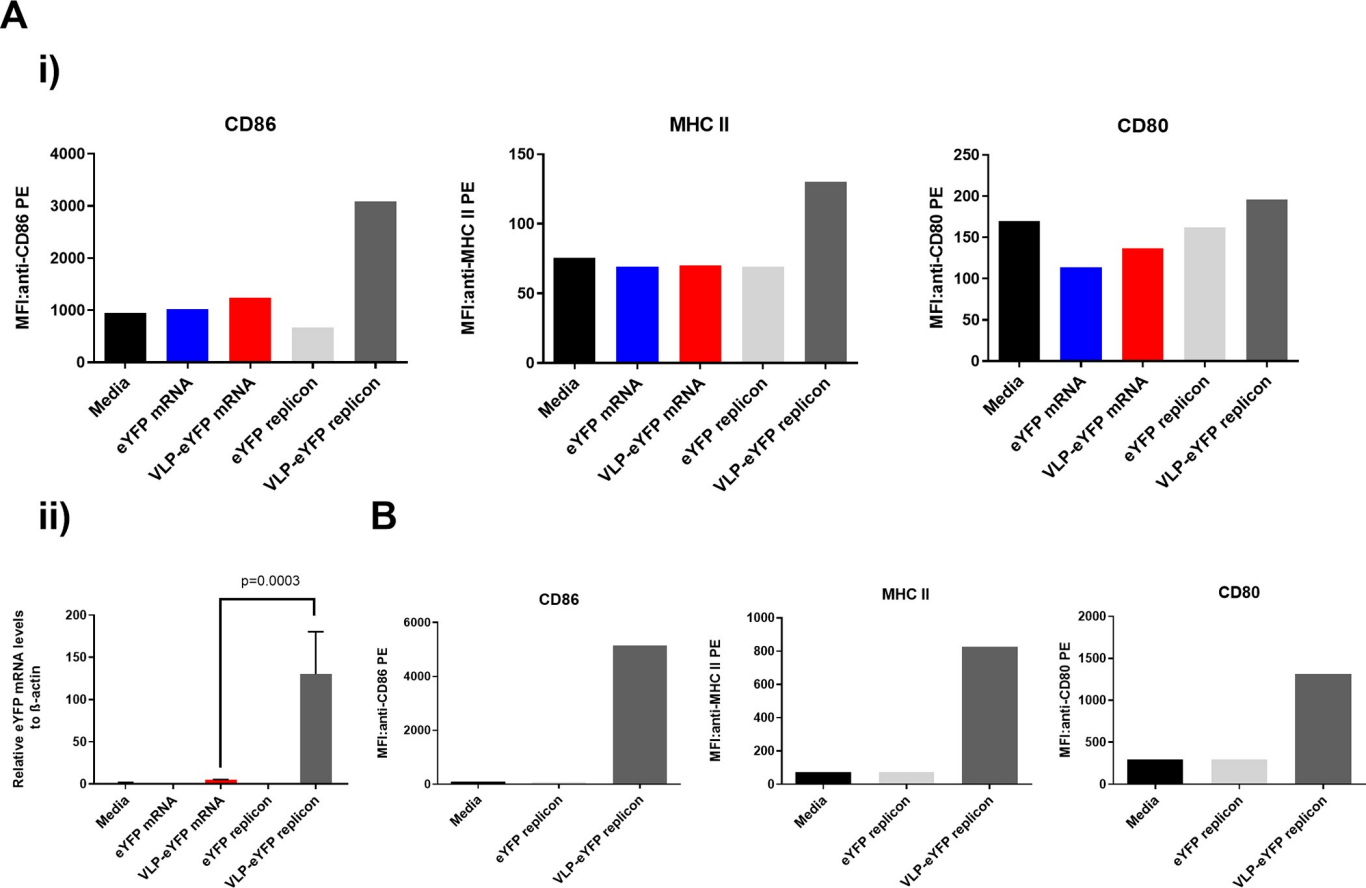
Expression intensity of activation markers on DCs. **A i)** Results from a donor treated with different conditions of EYFP mRNA or CCMV VLPs. The median fluorescence intensity (MFI) of CD86 (left), MHC II (middle) and CD80 (right) was determined by FACS assays for cells treated with media only (black bars), eYFP mRNA (blue bars), CCMV VLPs carrying eYFP mRNA (red bars), eYFP replicon mRNA (light grey bars), or CCMV VLPs carrying eYFP replicon (dark grey bars). **A ii)** RNA quantitative PCR (qPCR). The same-donor DCs treated/incubated with each of the five reagents in Ai were lysed after 24 hours and eYFP-specific mRNA levels were determined by qPCR. Their numbers relative to beta-actin multiplied by 1000 are plotted. From the same sample of dendritic cells assayed for maturation markers, triplicate measurements yielded the plotted average and standard deviation (p value was determined using a one-way Anova test). **B** Maturation markers probed as in Ai but with DCs from a different donor, for incubations with media only (black bars), eYFP replicon mRNA (light grey bars), and CCMV VLPs carrying eYFP replicon mRNA (dark grey bars).

Fig 5Ai shows panels of CD86, MHCII and CD80 marker activation, determined by flow cytometry, following incubation of immature DCs with medium, naked eYFP mRNA, CCMV VLPs carrying eYFP mRNA, naked eYFP replicon mRNA, and CCMV VLPs carrying eYFP replicon mRNA. Fig 5Aii shows the RNA levels corresponding to each of these five incubations, determined by qPCR, and plotted as numbers relative to those of the housekeeping gene beta-actin. Consistent with the well-known fact that DC maturation is extremely sensitive to culturing conditions, e.g., to medium and pipetting, marker activation by media is comparable to that by our “active” reagents, except for the case of replicon-containing VLPs. RNA replication – see Fig 5Aii – is present only for the replicon reagents and is 100 times greater for the packaged replicon. The sensitivity of DC maturation to donor cells is seen in Fig 5B, where DCs from a different donor than in 5A are used for incubations with medium (negative control), naked eYFP replicon mRNA, and CCMV VLPs carrying eYFP replicon mRNA. Here, while there is again comparable maturation associated with medium as with the naked replicon, the activation is one to two orders-of-magnitude greater for the VLP-packaged replicon.

### Pre-incubation of VLPs with VLP-antibodies enhances DC activation and RNA replication

To check whether antibodies against the CCMV VLPs can modulate the RNA uptake and activation of DCs, mice were immunized three times subcutaneously (see METHODS) with CCMV VLPs containing a non-translated RNA. Pre- and post-immune sera were collected and antibody titers were determined by ELISA against the CCMV VLPs. As shown in S1 Fig, strong antibody responses were observed in all of the animals.

**Fig 6:**
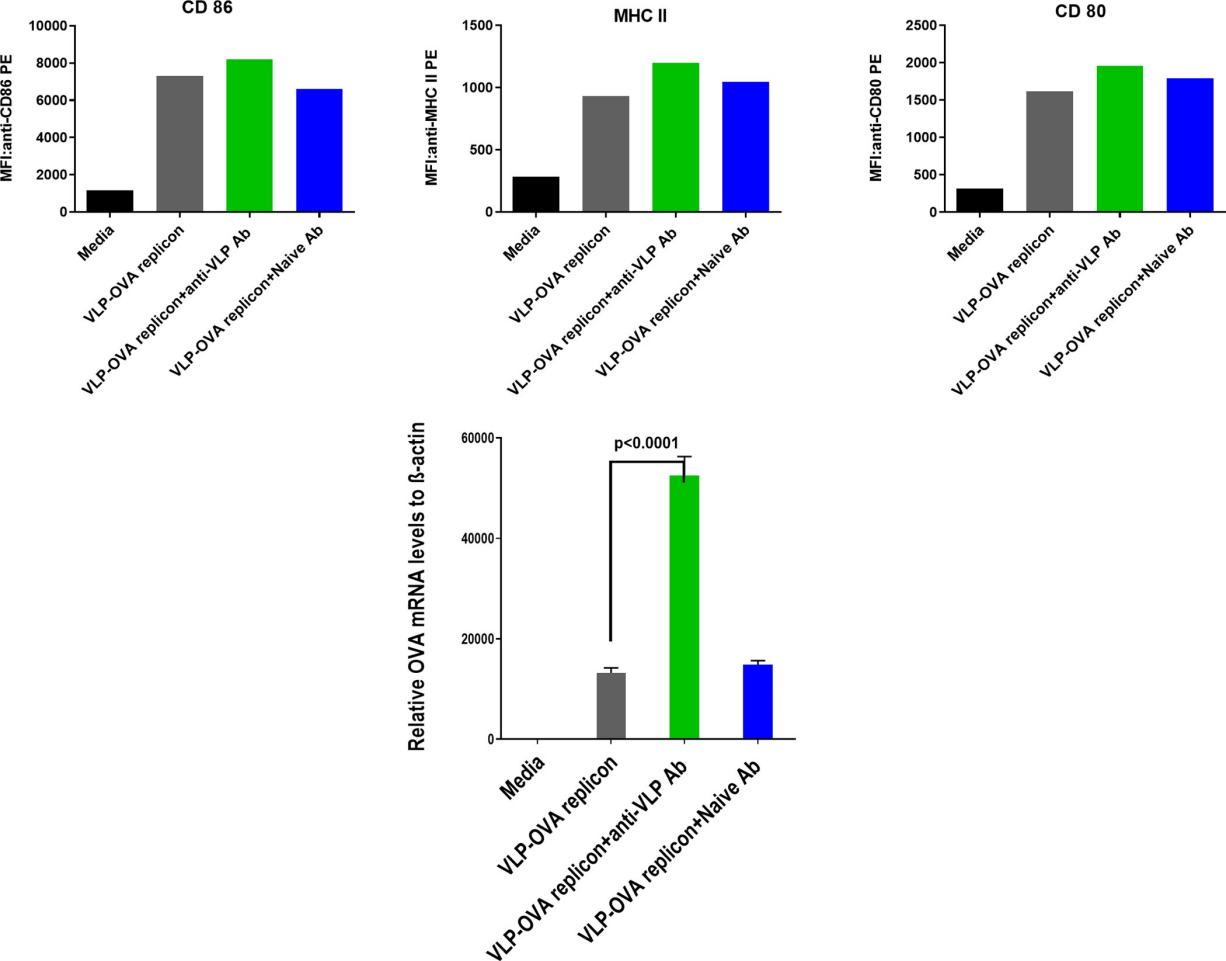
TOP: Expression intensity of activation markers on DCs treated with pre-incubated OVA-Replicon VLPs. The median fluorescence intensities (MFIs) of CD86 (left), MHC II (middle) and CD80 (right) were determined for cells treated with media only (black bars), CCMV VLPs carrying OVA replicon mRNA (grey bars), or CCMV VLPs carrying OVA replicon that have been pre-incubated with anti-CCMV VLP (green bars) or naïve serum antibodies (blue bars). **BOTTOM: RNA qPCR**. DCs treated with one or another of these three “reagents” were lysed and Ova-specific mRNA levels were determined by qPCR (again measured in triplicate as in Fig 5 Aii). The p value was determined using a one-way Anova test.

Fig 6 shows (top) the enhancement of DC marker activation when DCs are incubated with VLPs that have been exposed to capsid protein antibodies (in the form of serum from CCMV-inoculated mice). Here the packaged replicon includes the OVA tandem repeats, rather than eYFP. Again the enhanced marker activation associated with the pre-incubated VLPs correlates with a much larger enhancement in the level of RNA replication (see Fig 6 BOTTOM).

Similar results are obtained with the same donor cells for VLPs containing the eYFP replicon – see S2 Fig – i.e., marker activation is enhanced by pre-incubation with anti-CCMV VLP antibody, again associated with a much greater enhancement of RNA replication.

### Mouse vaccination induces specific T-cell response

**Fig 7:**
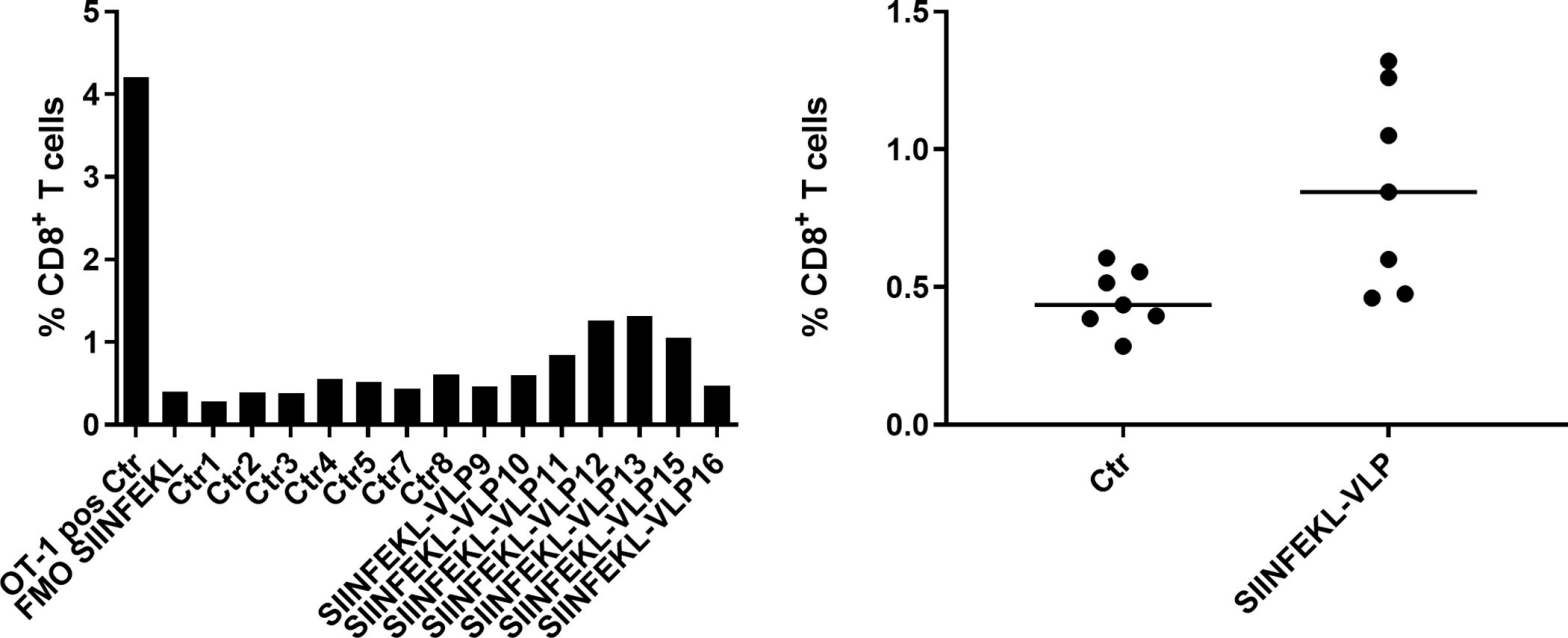
Percentage of SIINFEKL specific CD8^+^ T-cells from mice vaccinated three times, separated by one-week intervals. Cells isolated from spleens were analyzed ex vivo by flow cytometry one week after the last vaccination. LEFT: The positive control (“OT-1 pos Ctr”, left-most bar) is the result from a 4:96 mixture of splenocytes from an OT-1 mouse (homozygous for the transgenic T cell receptor recognizing SIINFEKL in the context of H-2Kb) and from a wildtype mouse; it provides a calibration of SIINFEKL-specific T-cell percentages harvested from the vaccinated mice. “FMO (fluorescence minus one) SIINFEKL” refers to a negative control in which the H-2Kb/SIINFEKL Pentamer is absent from the fluorochrome mix used for flow analysis. The “Ctr” measurements involve 7 wildtype mice (1-5, 7&8) vaccinated with buffer solution, while the“SIINFEKL-VLP” data refer to mice (9-13, 15-16) vaccinated with buffer with SIINFEKL replicon VLPs. RIGHT: The same data are presented as scatter plots, including the median (line) for each set of measurements. An unpaired t-test with Welch’s correction was performed and a significant difference between the groups was seen (p=0.0248).

In a preliminary *in vivo* study we vaccinated mice with in vitro reconstituted CCMV VLPs containing the SIINFEKL replicon RNA. Three subcutaneous (s.c.) injections consisting of 100μg of VLP in a 100μl volume were administered, separated by an interval of one week, and on the 4^th^ week ex vivo analyses of spleen T cells were carried out by FACS. The negative control experiment (“Ctr”) was a corresponding series of injections of buffer solution, and the positive control was the T-cell analysis of a 96:4 mixture of T-cells from wildtype mice and from OT-1 mice engineered with a SIINFEKEL-specific T-cell receptor. As seen in Fig 7 there is a small (0.4%) but significant T-cell response to the SIINFEKL replicon VLPs. Similarly, there is a small but significant increase in the percentage of T cells that are doubly positive for induction of both IFN γ and TNFα; see S3 Fig.

## Conclusions

We have shown that levels of DC activation are significantly enhanced by incorporation of reporter genes and model antigens into self-replicating RNA molecules – replicons – that have been *in vitro* packaged into VLPs. This enhancement of marker protein expression has been correlated with a much stronger amplification of RNA replication of the replicons, corresponding to RNA levels as much as ten-thousand-fold greater than those of the housekeeping protein beta-actin. Further enhancement of DC activation and RNA replication – likely facilitated by Fc- receptor-mediated[26] VLP uptake by DCs – has been achieved by pre-incubation of the VLPs with antibodies elicited against capsids of the proteins used to package the replicons. And this amplification is seen in dendritic cells from different donors and for different reporter and vaccine RNA genes. Preliminary in vivo experiments involving vaccination of mice with antigen-replicon VLPs show a small but significant generation of antigen-specific T cells that are doubly positive in their induction of IFN and TFN cytokines.

The modest T-cell response reported here provides a proof-of-principle for our RNA replicon virus-like particle vaccine platform. In contrast with plasmid-DNA and viral-vector delivery systems, in particular those involving lentiviruses, adenovirus, and adeno-associated virus, there is no need for genetic information to access the nucleus of the host cell and no possibility of integrating genes into the DNA of the host cell. There are also no viral-specific immune responses that interfere with efficacy of vaccine boosts. mRNA vaccines have none of these drawbacks because they are translated directly in the cytoplasm and gene expression is only transient. They also have the advantage that they act simultaneously as adjuvants, because they interact with pattern-recognition receptors that elicit innate immune response enhancing the maturation of DCs and induction of adaptive immune response. Further when combined with the right RNA replicase gene, the mRNA vaccine is amplified strongly before being translated, resulting in a high level of in situ protein expression. Several mRNA replicon vaccines of this kind have been reported (see the recent review by Maruggi, et al.[27], and references contained therein), but ours is the first in which the RNA is in vitro reconstituted in a perfectly monodisperse, RNase-resistant, spherical protein shell, instead of being “packaged” in less-well-characterized complexes involving lipids, cationic polymers, or various combinations of these components. In addition, our virus-like particles can be conjugated with ligands that enhance their uptake by targeted dendritic cells, and additional RNA genes included to further enhance dendritic cell activation and subsequent T cell priming.

The two essential ingredients of our approach derive from the special properties of two different non-mammalian viruses: (1) the insect virus Nodamura provides the self-replicating RNA molecule (with its associated RNA-dependent RNA polymerase) into which genes of interest (such as the OVA antigen) can be inserted; and (2) the plant virus CCMV provides the capsid protein ensuring the *in vitro* packaging of these replicons and consequent uptake by DCs. Nodamura is remarkable insofar as its RdRp functions in mammalian cells and is short enough to be packaged by CCMV CP, even after inclusion of interesting reporter genes or model antigens. And CCMV is remarkable insofar as its purified CP is uniquely capable of completely packaging, by spontaneous self-assembly, essentially any RNA sequence as long as its length does not exceed about 4000nt.

## Materials and methods

### Reagents

Recombinant human GM-CSF and IL-4 were purchased from R&D Systems (Minneapolis, MN, USA). PE-labeled antibodies (Abs) against human CD80, CD86 and MHC II were from BD Pharmingen (San Diego, CA, USA) and isotype control Abs from R&D Systems (Minneapolis, MN, USA). Complete Freund's Adjuvant, Incomplete Freund's Adjuvant and Lipopolysaccharide (LPS; Escherichia coli, O111:B4) were obtained from Sigma (St Louis, MO, USA). The goat anti-mouse HRP-conjugated secondary ab (goat anti-mouse IgG-HRP SC-2005, Lot # D1614) was purchased from Santa Cruz Biotechnology (San Diego, CA, USA). The BioFXr TMB super-sensitive one-component HRP microwell substrate was from SurModics (Minneapolis, MN, USA) and the BioStack Microplate Stacker from BioTek Instruments (Winooski, VT, USA). RPMI-1640 (-L glutamine) and L glutamine were from Thermo Fisher (Waltham, MA, USA) and heat-inactivated fetal bovine serum (FBS) from Sigma (F4135-500, 15H095H1).

### BHK cell culturing and transfections

BHK-21 cells were cultured in medium composed of high-glucose DMEM medium with 10% FBS and penicillin/streptomycin. The incubation conditions were 37˚C and 5% CO_2_. Standard passaging was performed with the involvement of PBS for washing, and 0.25% trypsin-EDTA for cell detachment. All the above cell culturing reagents and medium were from ThermoFisher. The cells were passaged to 60-90% confluence in 24-well cell-culture-treated plates and, prior to transfection, were washed with PBS. For each well, 5 μl of lipofectamine-2000 (Thermo Fisher) was diluted with Opti-mem medium (Thermo Fisher) before mixing with 0.5 μg of RNA that is also diluted to an equal volume in Opti-mem. Since the replicon is so effective, the masses of mRNA prescribed in the lipofectamine-2000 manufacturer’s protocol for the replicon were making the cells sick. By diluting the replicon RNA, a similarly large luciferase activity compared to higher concentrations of transfected mRNA is observed (especially when compared to the equimolar luciferase mRNA). Masses of 12.5 ng of luciferase replicon mRNA and 3 ng of luciferase mRNA were diluted with uncapped carrier RNA of similar length up to 0.5 μg, in order to achieve a similar number of liposomes per transfection.

### Luciferase assay

The luciferase assay was performed using the *Renilla* Luciferase Assay System from Promega (Fitchburg, WI, USA), and activity was measured using a Monolight 2010 luminometer. Cells were washed with PBS before directly lysing using passive lysis buffer and a cell scraper. 100 μl of *Renilla* luciferase assay reagent was added to a polycarbonate tube and luminescence was measured with 10 seconds of integration and a 2-second delay directly after adding 20 μl of cell lysate and mixing. Luciferase activity was performed in biological and assay duplicates for each time point.

### Cloning of replicon constructs

The plasmids constructed for these experiments were derived from plasmids kindly provided by Dr. Leonid Gitlin, from his work in Prof. Raul Andino’s lab at UCSF. Synthesis was performed from Nodamura virus strain Mag115, cDNA using random hexamers and SuperScriptIII (Invitrogen). The eYFP-replicon used was the same construct entitled pNoda-B1-FPG in their paper. The luciferase-replicon and OVA epitope-replicon plasmids were constructed from their construct pNoda-PolT2A-GFP. The luciferase gene for the luciferase-replicon was PCR-amplified from the plasmid pSP64-Ren Luc-Poly(A) – denoted as SP6 luciferase in Fig 3. pSP64-Ren Luc-Poly(A) has the Renilla luciferase open reading frame from pRL null (Progema), cloned into the XbaI site of pSP64 Poly(A) vector (Promega). The gene was PCR-amplified gene using Phusion High-Fidelity Polymerase (NEB), and included NdeI and AgeI restriction sites for insertion into the corresponding sites of the pNoda-PolT2A backbone. The construction of the OVA epitope-replicon plasmid began with ordering complementary oligos encoding the SIINFEKL peptide sequence from IDT. These oligos have flanking NheI and XbaI sequences, as well as an overhanging ‘A’ nucleotide at the 3’ ends to allow for ‘TA-cloning’ into a pGEM-T Easy Vector (Promega). When the pGEM vector with inserted SIINFEKL sequence is digested with a combination of either ScaI/XbaI or ScaI/NheI, the remaining XbaI and NheI sites are complementary. This process is repeated to result in a 4X tandem OVA epitope sequence, which is then cloned into the pNoda-PolT2A backbone by the same methods as the luciferase-replicon.

### Synthesis of replicon mRNA-VLPs

The replicon constructs were linearized with XbaI and subsequently purified using QIAquick PCR purification columns from Qiagen (Valencia, CA, USA). The linearized DNA was transcribed with T7 RNA polymerase. The buffer composition of the reaction was 40 mM Tris pH 7.5, 25 mM MgCl_2_, 4 mM μl DTT, and 2 mM spermidine. The rNTP concentration was 7.5 mM. Incubation was for 3 hours at 37°C, followed by a 30-minute incubation with turbo DNase at the same temperature. The resulting RNA was purified using the RNeasy mini kit from Qiagen, and then capped using the Vaccinia Capping System from New England Biolabs (Beverly, MA, USA). This process added a 7-methylguanylate cap structure to the 5’ end of RNA in the presence of the capping enzyme, reaction buffer, GTP, and the methyl donor, SAM. The *in vitro* transcription and capping were both done in the presence of RNase inhibitors to preserve the RNA, and the capping mixture was again purified by an RNeasy mini column (Qiagen). Generating VLPs first involves isolation of CCMV CP from CCMV. The CCMV was purified from infected Cowpea plant leaves. To purify the CP, the virion was loaded into 6 kDa dialysis bags, and dialyzed in disassembly buffer (500 mM calcium chloride, 50 mM Tris-HCl pH 7.5, 1 mM EDTA, 1 mM DTT, and 0.5 mM PMSF). After dialysis, the disassembled CP-RNA mixture was pipetted into polycarbonate tubes and centrifuged in a TLA 110 rotor at 95k for 2 hours. (This dialysis step and the three described immediately below – for CP purification, CP-RNA binding, and capsid formation – were each carried out for 6 hours at 4°C.) Fractions were taken after centrifugation, and a threshold A260/280 ratio of 0.6 was used to verify that they did not contain RNA. The capsid protein was dialyzed into Buffer B (1 M NaCl, 50 mM Tris-HCl pH 7.2, 1 mM EDTA, 1 mM DTT, and 1 mM PMSF). After determining the CP concentration with a Nanodrop Spectrophotometer, CP was combined with mRNA at a ratio of 12.9 μg of CP to 3μg mRNA (representing the minimum excess of CP necessary for complete encapsidation of mRNA) in buffer B but without DTT or PMSF, for a total volume of 100μl. This mixture was then dialyzed into RNA Assembly Buffer, RAB (50 mM NaCl, 50 mM Tris-HCl pH 7.2, 10 mM KCl, 5 mM MgCl_2_, and 1 mM DTT), after which the pH was dropped to form capsids by dialyzing into Virus Suspension Buffer (50 mM Sodium Acetate, 8 mM Magnesium Acetate). Finally, the sample was transferred to RAB without DTT in order to have a VLP sample at the necessary neutral pH for biological experiments. Once VLPs have been verified (methods for which are described in the following two sections), the mg/ml concentration of VLPs is measured using the Nanodrop by dividing the 260nm absorbance by 5.8, the same factor used when determining the concentration of purified virus. Because of the small excess of CP used for complete packaging of RNA, the recovery of VLP mass is about 80% of the total mass of mRNA and CP used in the assembly.

### Verification of assembly

After assembly, the first step to verify the formation of homogeneous VLPs is agarose gel electrophoresis. A native agarose gel was run at 50 V for 1.5 hours, with the running buffer and the 1% agarose gel being made of 100 mM Sodium Acetate and 1 mM EDTA; the staining is with ethidium bromide. For reference, wild-type CCMV virion is included in a separate lane – the middle, in Fig 4A. The assembly mix is treated with RNase and loaded directly onto the gel (rightmost lane): the reconstituted VLPs run more slowly because of excess protein adsorbed onto their capsids; a small (40%) excess of capsid protein has been used to guarantee complete packaging of all the RNA, and it binds to the negative exteriors of the self-assembled VLPs through its cationic N-terminus[18–21]. VLP formation from in vitro assemblies has also been verified directly in bulk solution by sucrose gradient analysis (see our earlier study of VLPs involving the same lengths of RNA[20] – in particular Fig 3 there), where we compare the relative sedimentation profile peak positions for VLPs, wild-type virions, and virions plus excess protein and correlate them with the corresponding band positions in the electrophoretic gels.

### Electron microscopy

The preparation of well-formed intact VLPs was confirmed by negative-stain electron microscopy. 6 μl of VLPs is applied to glow-discharged copper grids (400-mesh) coated with Parlodion and carbon. After 1 min, the solution is blotted with Whatman filter paper. 6 μl of 1% uranyl acetate is then added to the grid. After 1 min, the excess uranyl acetate is removed by blotting. The grids are stored overnight in a desiccator and analyzed with a JEM 1200-EX transmission electron microscope equipped with a wide-angle (top mount) BioScan 600-W 1×1K pixel digital camera operated at 80 keV. The diameter of the VLPs is estimated by taking the geometric mean of two orthogonal measurements of the capsids obtained with ImageJ (U.S. National Institutes of Health) software from recorded images. The diameter of 100 VLPs is measured in this way and compared to the diameter of wtCCMV that had been previously imaged.

### Generation and culture of monocyte-derived DCs

To generate immature DCs, CD14^-^ monocytes negatively isolated from human peripheral blood mononuclear cells were cultured in a 24-well plate (1.0 × 10^6^ cells/ml/well) with RPMI-1640 (-L glutamine) medium containing 1 × L glutamine and 10% heat-inactivated fetal bovine serum for 5 days. The donors were anonymous, but the number designation of PBMC for the figures included in this article are as follows: Fig 5Aiand 5Aii – A5394, Fig 5B – A5154, Fig 6 – A5167, and S2 Fig – A5167. Culture medium was supplemented with recombinant human GM-CSF (10 ng/mL) and recombinant human IL-4 (10 ng/mL).

### Pulsing of dendritic cells

On day 5, immature DCs (1.0 × 10^6^ cells/ml/well) were pulsed with naked eYFP mRNA, CCMV VLPs carrying eYFP mRNA, naked eYFP replicon mRNA, CCMV VLPs carrying eYFP replicon mRNA, CCMV VLPs carrying OVA-replicon mRNA, OVA-replicon-carrying CCMV VLPs pre-incubated with anti-CCMV VLP abs or naïve serum abs, and eYFP-replicon-carrying CCMV VLPs pre-incubated with anti-CCMV VLP abs or naïve serum abs. DCs stimulated with LPS and media were used as a positive and negative control, respectively. Following 24 hours of incubation, expression of activation markers for human DCs such as CD80, CD86 and MHC II was analyzed using flow cytometry.

### Mouse immunization

C57BL/6Tac mice between 6-8 weeks of age were purchased from Charles River Laboratories (Wilmington, MA). They were immunized subcutaneously with CCMV VLPs containing a non-translated RNA (20 μg/per mouse/per immunization) on weeks 0, 2, and 4 using Complete Freund's Adjuvant for priming and the subsequent boost with Incomplete Freund's Adjuvant. The animal use protocol was approved by IACUC committee of Boehringer Ingelheim Pharmaceuticals. Serum samples collected one week after the 3^rd^ immunization were evaluated by ELISA to determine the antibody titer against CCMV VLPs. Pre-bleed was used as a negative control.

### Enzyme-linked immunosorbent assay (ELISA)

To determine the end-point titers, CCMV VLPs were coated onto Immulon 2 HB 96-Well Microtiter plates (Catalog # 227, ImmunoChemistry Technologies, Bloomington, MN) at 0.5μg/mL using PBS overnight at 4°C. Uncoated surface was blocked using phosphate-buffered saline (PBS) containing 3% Bovine Serum Albumin for 1 hr at 37°C. The plates were subsequently washed 5× with 0.05% Tween 20 in PBS. Mouse serum serially diluted (three-fold) in the blocking buffer was added to each well and incubated for 2 hrs at 37°C. The plates were washed 5×, and horseradish peroxidase (HRP)-conjugated secondary ab (goat anti-mouse IgG, 1:3000 dilution) was added to each well and incubated for 1 hr at 37°C. Plates were washed 5× and developed by adding 100 μl BioFXr TMB super-sensitive one-component HRP microwell substrate for 5 min. Reactions were stopped with 50 μl of 2 N H_2_SO_4_. Plates were read on a microplate reader (BioStack Microplate Stacker) at 450 nm. All experiments were performed in duplicate.

### Quantitative PCR

The Power SYBR Green Cells-to-CT kit was used for the detection of OVA and eYFP mRNA uptake by DCs. Briefly, the passively pulsed dendritic cells were washed twice with ice-cold 1 × PBS. RNALater (200 μl) was added to the well(s). The plate was sealed with PetriSEAL and placed at -80^o^C. To isolate RNA, the plate was thawed rapidly and an equal volume of ice-cold 1 × PBS was added to the well(s). The plate was spun at 1400 rpm for 5 min at 4°C. The fluids were removed carefully and the cell layer was washed again twice with ice-cold 1 × PBS. The cells were lysed for 5 min at room temperature in 50 μl of Lysis solution supplemented with DNase I (all supplied in the Power SYBR Green Cells-to-CT kit). The reactions were then stopped with 1/10 volume stop solution and left at room temperature for 2 min. 10 μl of the lysate was added to the reverse transcription reaction cocktail that contained 1× SYBR RT buffer with 1× RT Enzyme mix. The reaction was run at 37°C for 1 hr followed by a 5-min 95°C heat kill of the enzyme.

Four microliters of the reverse-transcribed material was added to 16 μl of gene-specific forward and reverse primers at 400 nM in the PowerSYBR Green PCR master mix. The samples were run using a Viaa7TM cycler (95°C for 10 min to activate the enzyme; 40 cycles of 95°C for 15 seconds and 60°C for 1 minute to amplify the sequence of interest). The CT values were generated by the Viaa7 program. Relative expression of the pulsed mRNA was calculated by finding the differences between pulsed mRNA CT values and the CT value for the ACTB housekeeping gene generated from the same cells by using the relation: RelExp = POWER (2 -(CT value)).

For quantifying luciferase RNA in BHK cells in the Fig 3 time course, a different qPCR approach was utilized. The transfected BHK cells were lysed using buffer RLT in the RNeasy mini kit from Qiagen. Before purifying with the standard RNeasy mini kit protocol, the lysate was passed through a QIAshredder column (Qiagen) in order to remove insoluble debris. After purification, the concentration was verified with a Nanodrop Spectrophotometer, and 1 μg was reverse transcribed using M-MuLV reverse transcriptase from NEB. The flanking luciferase qPCR primer was used to synthesize the cDNA. In order to quantify RNA number, a standard curve with known concentrations of luciferase replicon RNA from 100 pg to to 10 fg was assayed by qPCR using the standard protocol for SsoAdvanced Universal SYBR Green Supermix (Bio-Rad, Irvine, CA, USA). The concentration of the luciferase amplicon primers used was 200 nM. For the time course samples, 100 pg and 10 pg cDNA of the luciferase mRNA and replicon transfected cells were assayed by qPCR, respectively.

### Mouse vaccinations

C57BL/6Tac female mice were provided by Taconic Denmark. The mice were transferred to the local AAALAC accredited animal facility at Boehringer Ingelheim RCV GmbH & Co KG at an age of 6-8 weeks, and immediately after arrival were allowed to adjust to conditions for at least 5 days before they were used for experiments. Standardized diet (PROVIMI KLIBA) and autoclaved tap water were provided *ad libitum*. Mice were group-housed (8 mice per cage) under pathogen-free and controlled environmental conditions (21 ± 1.5°C temperature, 55 ± 10% humidity) and handled according to the institutional, governmental and European Union guidelines (Austrian Animal Protection Laws, GV-SOLAS and FELASA guidelines). Animal studies were approved by the internal ethics committee and the local governmental committee.

Two different vaccination schedules were tested. In the first scenario mice were vaccinated subcutaneous (s.c.) only once, with either buffer as control, with the Ova Protein (50μg), with the SIINFEKL Replicon (100μg), or with the VLP containing the SIINFEKL replicon (100μg). The volume of all applications was 100μL, and after one week the mice were euthanized and spleens were harvested for FACS analysis. In the second vaccination scenario mice were vaccinated with buffer as control or with buffer containing VLPs containing the SIINFEKL replicon RNA (100μg). The vaccinations were administered s.c. with a volume of 100μL for 3 consecutive weeks (q7d), and one week after the last injection the mice were sacrificed and their spleens were harvested for FACS analysis. In addition one C57BL/6Tg(TcraTcrb)1100Mjb/Crl (OT-1) mouse provided by Charles River Laboratories (France) and one C57BL/6Tac mouse were euthanized and their spleens harvested to collect T-cells as control for the FACS analysis.

### *In vitro* stimulation and single-cell analysis by flow cytometry

Splenocytes from vaccinated and control mice were obtained by tissue homogenization followed by red blood cell lysis (ammonium-chloride-potassium lysis). Ovalbumin specific CD8^+^ T cells, present in the cell suspensions, were stimulated with 8μg/ml of SIINFEKL(Ova 257-264, InvivoGen) and TEWTSSNVMEERKIKV(EMC microcollections) peptides together with 10μg/ml anti-CD28 antibody (BioLegend) for 6 hours. One hour after start of stimulation, a protein-transport-inhibitor cocktail (eBioscience), containing Brefeldin A and Monensin, was added to block cytokine release. Before surface-marker staining, dead cells were labeled with a fixable viability stain and Fc receptors were blocked (TruStain fcX, BioLegend) to avoid nonspecific binding. Key surface markers were stained with fluorescently labeled antibodies, followed by fixation/permeabilization (BD Cytofix/Cytoperm Plus, BD Biosciences) and intracellular cytokine staining (FITC anti-mouse IFNγ and PE-Cy7 anti-mouse TNFα, eBioscience).

For immunophenotyping including the detection of H-2Kb/SIINFEKL specific CD8 T cells, spleens were dissociated using a spleen dissociation kit from Miltenyi biotec, together with a gentle MACS Dissociator. After red blood cell lysis, labeling of dead cells and Fc Receptor blockade, single cell suspensions were incubated with H-2Kb/SIINFEKL pentamer (ProImmune) for 30 minutes at 4 to 8^o^C. After thorough removal of unbound pentamer, key surface markers were labeled fluorescently with specific antibodies.

Flow data were collected with a BD LSRFortessa cell analyzer and further analyzed using FlowJo 10 and GraphPad Prism 7.03.

## Supporting information

S1 Fig Antibody titers. Antibody titers from mice immunized with CCMV VLPs containing a non-translated RNA.
(PDF)

S2 Fig Expression intensity of activation markers on DCs treated with CCMV VLPs carrying eYFP-Replicon mRNA and RNA quantitative PCR (qPCR). (PDF)

S3 Fig Cytokine production of SIINFEKL responsive CD8^+^ T cells. Amount of INFγ and TNFα double-positive cells when stimulated with SIINFEKL peptide.
(PDF)

S4 Fig Low frequency of H-2Kb/SIINFEKL-specific CD8^+^ T cells in mice vaccinated only one time. (PDF)

S1 File SI raw data and calculations for Figs 3, 5, 6, 7, S1, S2, S3 and S4. (XLSX)
