# Supplementary material for: Delivery of self-amplifying RNA vaccines in in vitro reconstituted virus-like particles
Source: PLoS One. 2019 Jun 4;14(6):e0215031. doi: 10.1371/journal.pone.0215031 (PMC6548422; doi:10.1371/journal.pone.0215031)
Supplement: S1 Fig — Antibody titers from mice immunized with CCMV VLPs containing a non-translated RNA. (PDF) [file pone.0215031.s001.pdf]

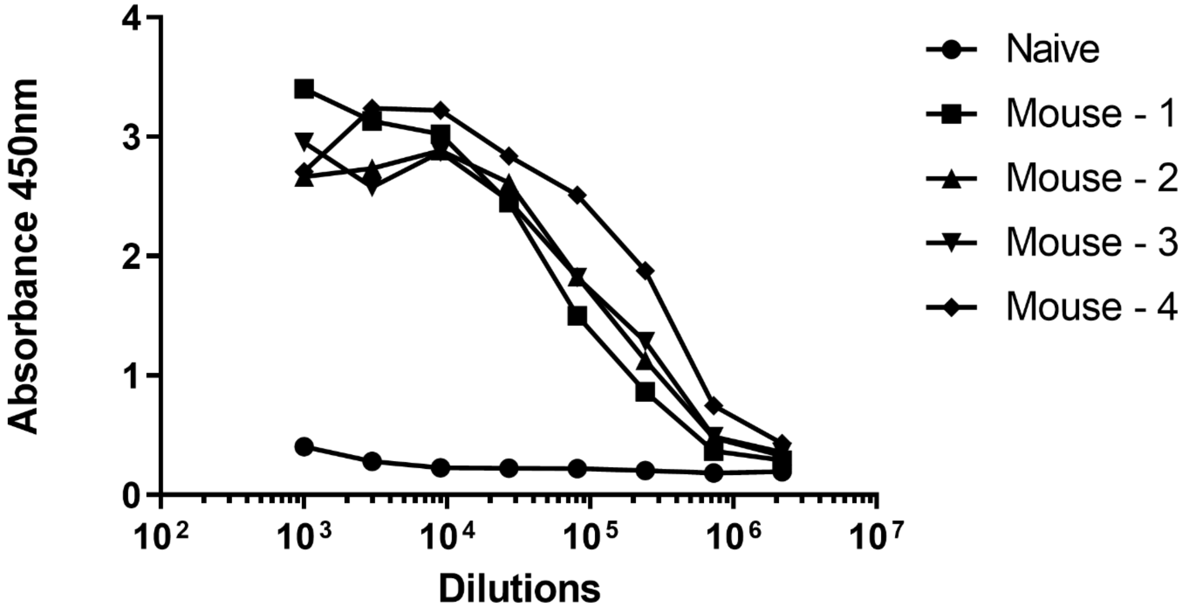

Figure S1. Antibody Titers. Four C57BL/6Tac mice were immunized subcutaneously with CCMV VLPs containing a non-translated RNA (20 µg/per mouse/per immunization) on weeks 0, 2, and 4. Sera were tested for binding to CCMV VLPs 1 week after the 3rd immunization. Pre-immune serum ("Naïve") was used as a negative control.
