# Supplementary material for: Delivery of self-amplifying RNA vaccines in in vitro reconstituted virus-like particles
Source: PLoS One. 2019 Jun 4;14(6):e0215031. doi: 10.1371/journal.pone.0215031 (PMC6548422; doi:10.1371/journal.pone.0215031)
Supplement: S2 Fig — (PDF) [file pone.0215031.s002.pdf]

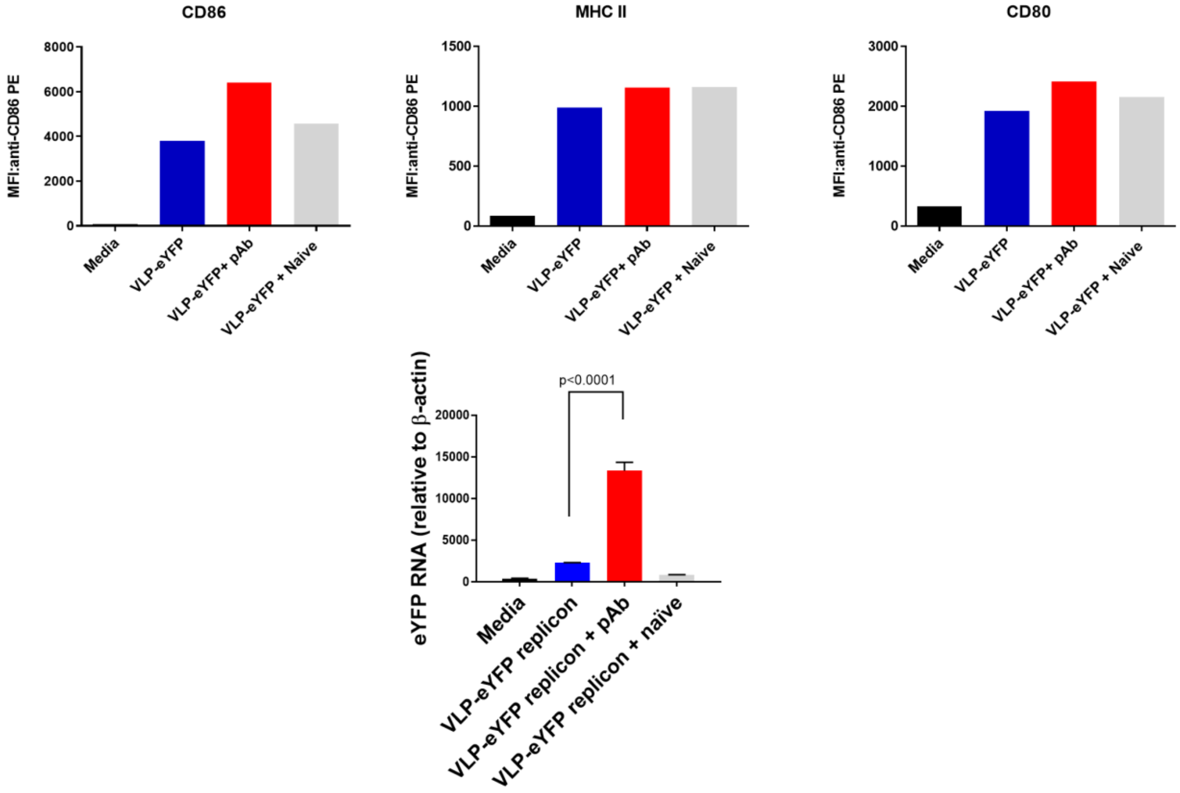

Figure S2. TOP: Expression intensity of activation markers on DCs treated with CCMV VLPs carrying eYFP-Replicon mRNA. The median fluorescence intensity (MFI) of CD86 (left), MHC II (middle) and CD80 (right) was determined for DCs treated with media only (black bars), CCMV VLPs carrying eYFP replicon mRNA (blue bars), and CCMV VLPs carrying eYFP replicon mRNA that have been pre-incubated with anti-CCMV VLP (red bars) antibodies or naïve antibodies (grey bars).
BOTTOM: RNA quantitative PCR (qPCR). DCs treated as above were lysed and eYFP-specific mRNA levels were determined by qPCR – note break in vertical scale. The p value was determined using a one-way Anova test.
