# Supplementary material for: Delivery of self-amplifying RNA vaccines in in vitro reconstituted virus-like particles
Source: PLoS One. 2019 Jun 4;14(6):e0215031. doi: 10.1371/journal.pone.0215031 (PMC6548422; doi:10.1371/journal.pone.0215031)
Supplement: S3 Fig — Amount of INFγ and TNFα double-positive cells when stimulated with SIINFEKL peptide. (PDF) [file pone.0215031.s003.pdf]

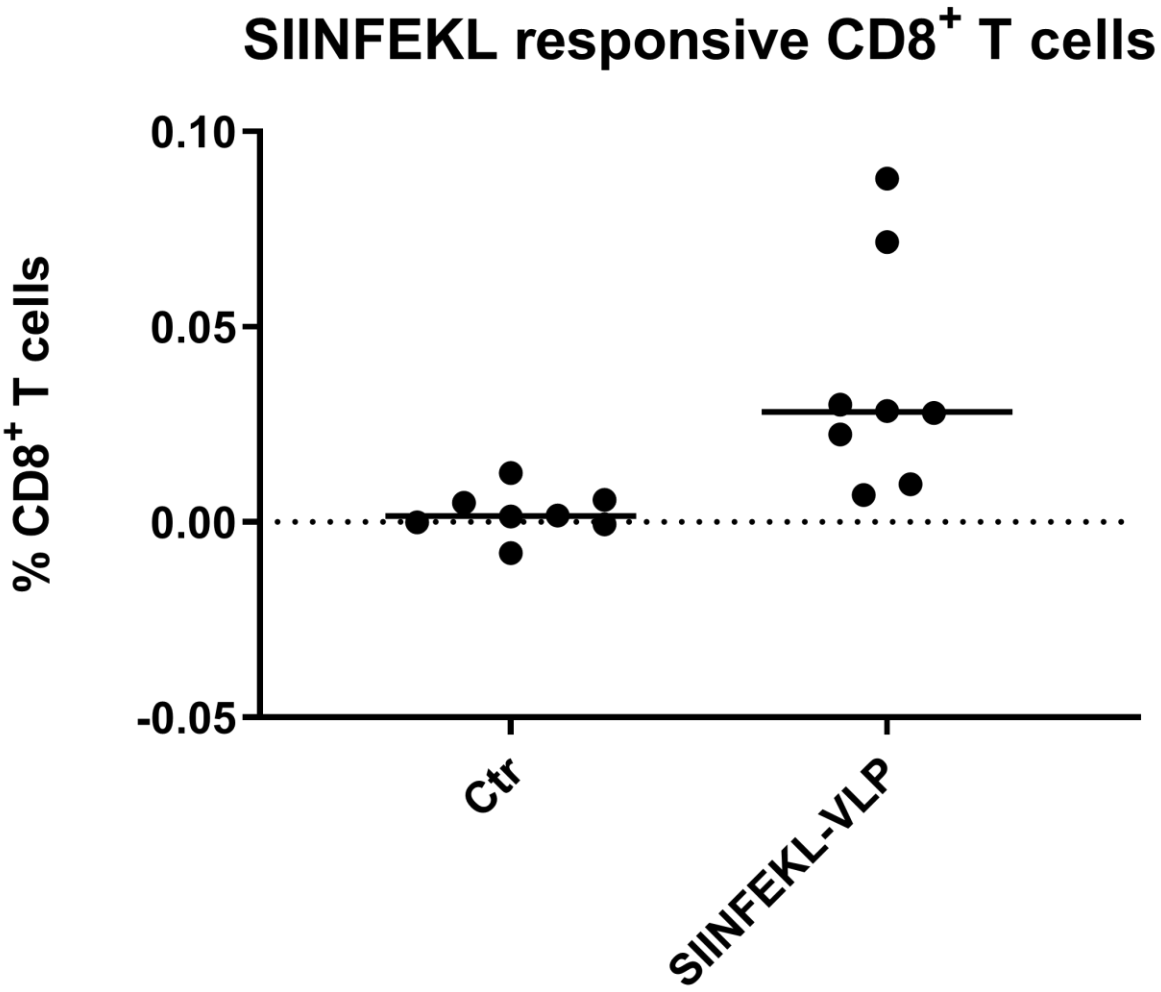

Figure S3. Cytokine production of SIINFEKL responsive CD8$^+$ T cells. T-cells were stimulated with SIINFEKL peptide for 6 hours. The amount of INFγ and TNFα double-positive cells is shown as percentage of total CD8$^+$ T cells. Samples analyzed in this assay have been obtained from the same animal experiment described in Figure 7, with “Ctr” and “SIINFEKL-VLP” denoting the multiple vaccinations involving buffer-solution and OVA-replicon-VLP, respectively.
