# Supplementary material for: Delivery of self-amplifying RNA vaccines in in vitro reconstituted virus-like particles
Source: PLoS One. 2019 Jun 4;14(6):e0215031. doi: 10.1371/journal.pone.0215031 (PMC6548422; doi:10.1371/journal.pone.0215031)
Supplement: S4 Fig — (PDF) [file pone.0215031.s004.pdf]

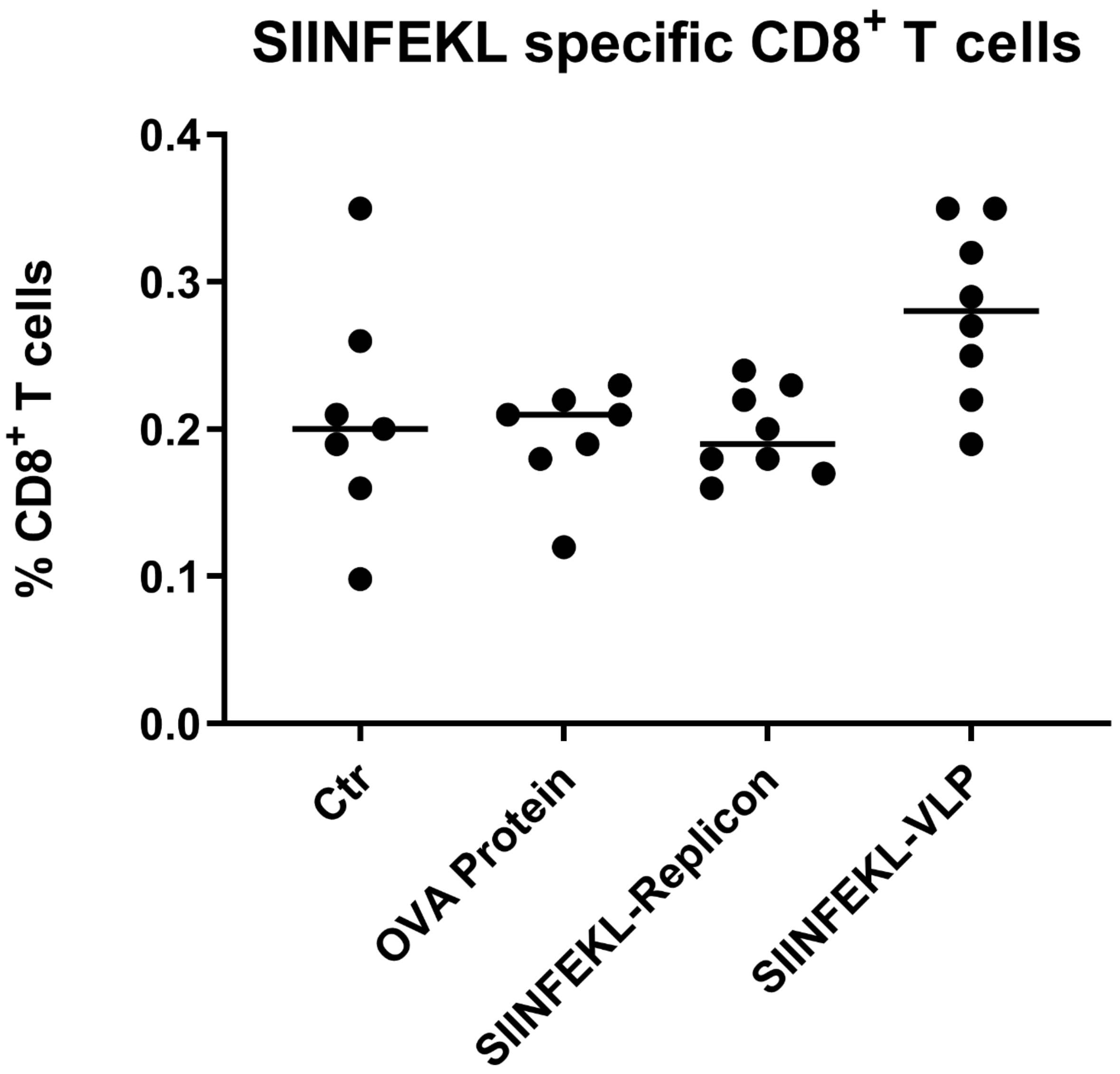

Figure S4. Low frequency of H-2Kb/SIINFEKL-specific CD8^{+} T cells in mice vaccinated only one time. The fraction of SIINFEKL specific T-cells in spleen samples, collected one week following a one time only vaccination, was determined by flow cytometry. The graph shows scatter plots for single vaccinations with negative control ("Ctr", 100 µL of buffer solution) positive control (50 µg of "OVA Protein"), 100 µg of SIINFEKL replicon RNA and 100 µg of SIINFEKL-VLPs. The difference of less than 0.1% between the SIINFEKL-VLP outcome and that for the negative control is to be contrasted with the 0.4% difference found for the multiple-boost vaccination with the same VLPs (see Figure 7). To compare all groups an Anova test was performed together with a Dunnett's test to correct for multiple comparisons (p= 0.0484) between the "Ctr" and the "SIINFEKL-VLP" groups.
